# Complete response to encorafenib plus binimetinib in a *BRAF V600E*-mutant metastasic malignant glomus tumor

**DOI:** 10.18632/oncotarget.28654

**Published:** 2024-10-11

**Authors:** Marta Arregui, Antonio Calles, María del Mar Galera, Ana Gutiérrez, Carlos López-Jiménez, Carolina Agra, Adriana Fernández, Natalia Gutiérrez, María de Toro, Rosa Álvarez

**Affiliations:** ^1^Department of Medical Oncology, Gregorio Marañón University Hospital, Madrid 28007, Spain; ^2^Department of Medical Oncology, Fundación Jiménez Díaz University Hospital, Madrid 28040, Spain; ^3^Department of Pathology, Gregorio Marañón University Hospital, Madrid 28007, Spain; ^4^Department of Radiology, Gregorio Marañón University Hospital, Madrid 28007, Spain

**Keywords:** malignant glomus tumor, glomangiosarcoma, BRAF V600E, agnostic treatment, targeted therapy

## Abstract

Glomus tumors (GT) are very rare mesenchymal neoplasms arising from glomus bodies, arteriovenous structures located in the dermis and involved in thermoregulation. Although most are benign, they may occasionally present malignant histological features associated with aggressive clinical behavior, metastatic spread, and poor response to conventional chemotherapy. The BRAF V600E mutation has been identified in a subset of malignant GT, highlighting a promising therapeutic target. Here, we report the impressive clinical and morpho-metabolic response of a metastatic BRAF V600E-mutated glomangiosarcoma after treatment with encorafenib and binimetinib.

## INTRODUCTION

Glomus tumors (GT) are rare mesenchymal neoplasms that constitute less than 2% of soft tissue tumors and arise from structures called glomus bodies [1]. These structures are neuromyoarterial systems responsible for the regulation of local blood flow and thermoregulation, located in the deep dermis of any part of the body, with a greater concentration on hands and feet. Up to 80% of GT are in the upper extremities and are especially frequent in the subungual region. The classic clinical presentation is the triad of paroxysmal pain, cold intolerance, and point tenderness. Most are diagnosed in young adults (20–40 years) although they can occur at any age and with equal frequency in men and women [2].

Histologically, GT are well-circumscribed lesions with capillary vessels surrounded by glomus cells in a hyalinized or myxoid stroma. Although most of them are benign, some rare examples show histologic malignant features associated with aggressive clinical behavior and a poor prognosis in advanced disease. WHO criteria for malignant GT are defined by marked nuclear atypia and any grade of mitotic activity, or atypical mitotic figures. Tumors larger than 2 cm and with a deep location are usually considered malignant as well. Those with only one atypical feature are classified as GT of uncertain malignant potential. Pathology of malignant GT shows either a malignant spindle-shaped morphology or round cells with malignant characteristics [1, 2].

BRAF V600E mutations have been reported in approximately 10% of sporadic GTs [3] and have been found to be associated with atypical characteristics, such as large size, deep location, infiltrative behavior, and pathology of malignant or uncertain malignant potential, indicating a more aggressive clinical course and potential for metastatic spread [3–8]. Surgery is the preferred treatment for practically all patients with localized disease [2–4, 6–8]. Nevertheless, for those with unresectable tumors, unacceptable postoperative morbidity, recurrence after resection, or metastatic disease, systemic therapy is advised. In these cases, the presence of BRAF V600E targeted therapy could be a promising therapeutic option, as first described by Cuviello et al., who successfully used BRAF/MEK targeted therapy to treat a BRAF V600E-mutant localized malignant GT of the brachial plexus [5].

We present the case of a 45-year-old male patient diagnosed with stage IV malignant GT harboring a BRAF V600E mutation. The patient received systemic treatment with encorafenib in combination with binimetinib, resulting in a rapid clinical and radiological response.

## CASE REPORT

A 45-year-old male, with no medical or family history of interest, developed post-traumatic, stabbing pain in the right intercostal area, at the age of ten years. A biopsy was performed without a pathological diagnosis of malignancy.

**Figure 1:**
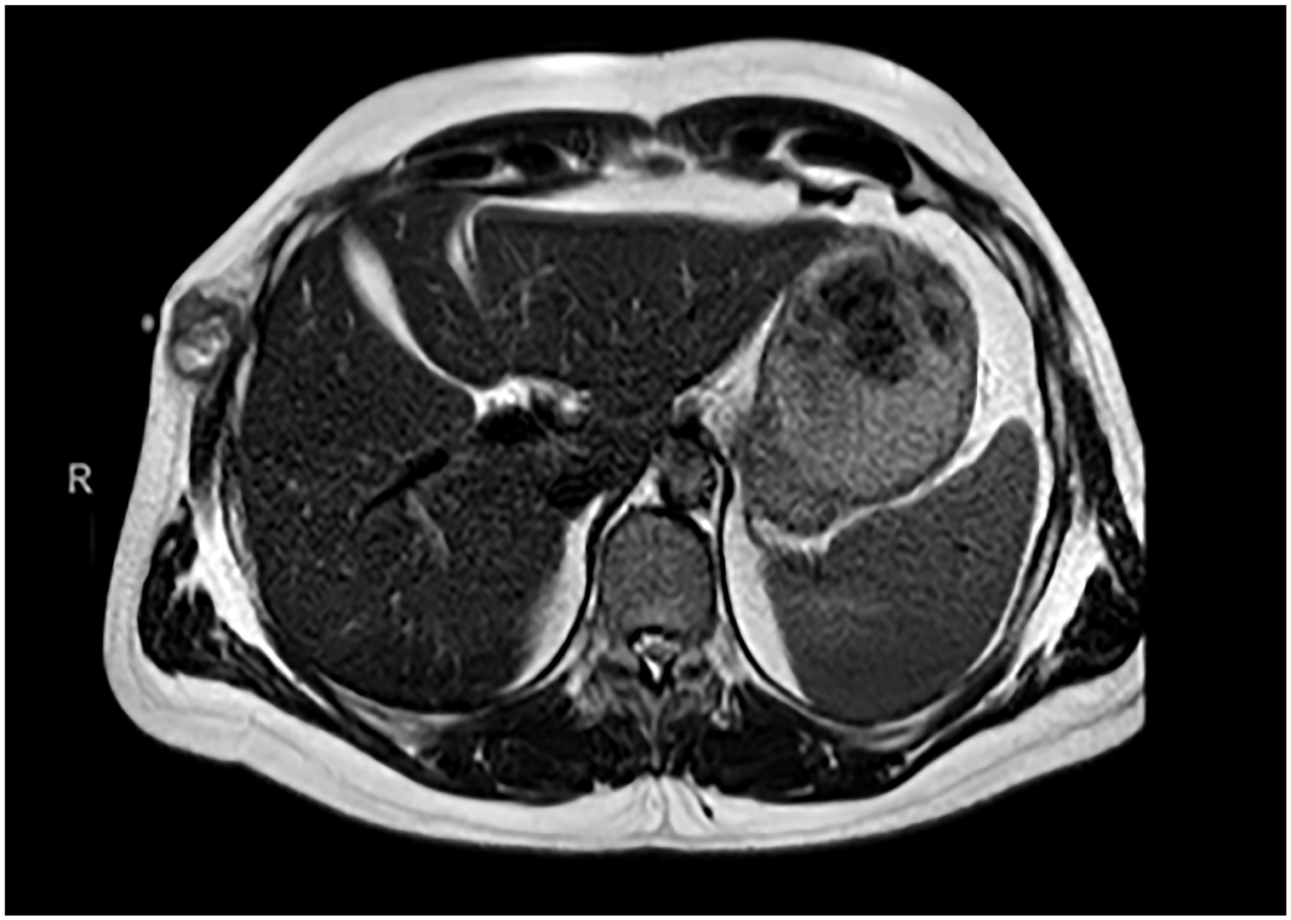
Chest MRI 04/22/2021. Soft tissue tumor (32 × 23 mm) on the right chest wall, located deep to the superficial fascia, in relation to the serratus muscle and extending towards the intercostal musculature, superficial to the costal arches. Small subcutaneous tumors, the largest measuring 8 mm in diameter, on the thoracic and abdominal wall.

25 years later, in April 2021, due to persistence of pain and the appearance of a palpable mass at that level, magnetic resonance imaging (MRI) was performed, showing a tumor located in the right chest wall, extending towards the intercostal musculature and accompanied by two small adjacent subcutaneous tumors (Figure 1).

**Figure 2:**
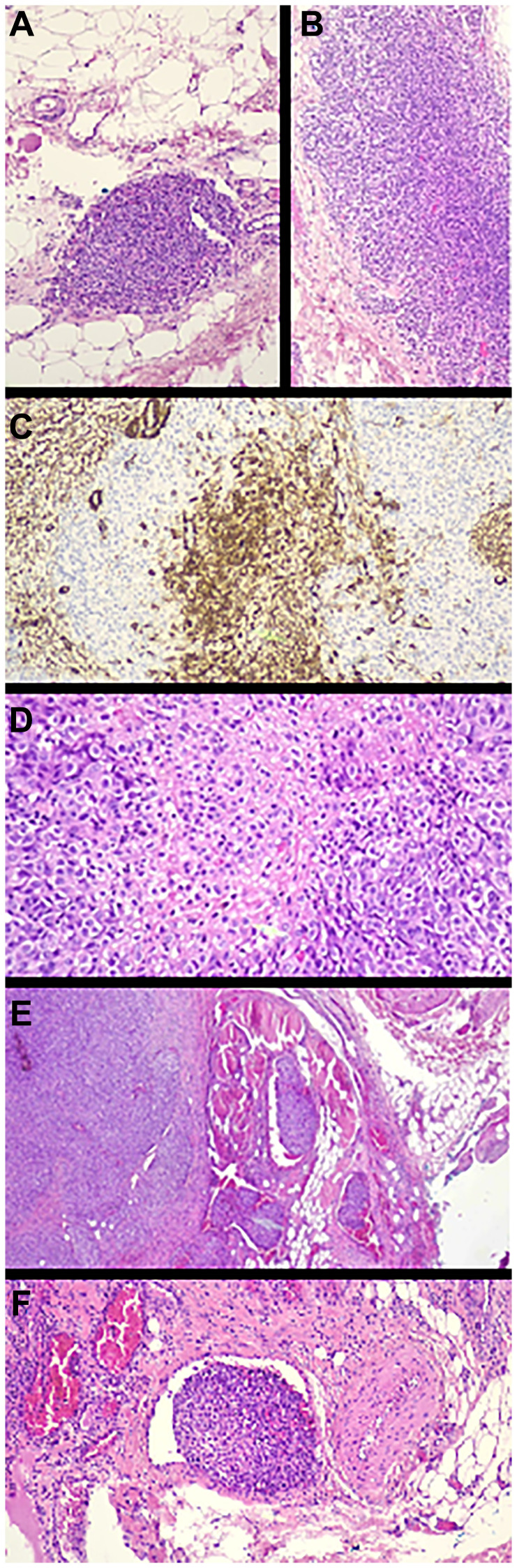
Pathology images (**A**–**D**). Immunohistochemistry staining. Histologically, a well-demarcated, unencapsulated, multinodular, densely cellular neoplasm is observed, that grows forming large nodules composed of cells of epithelioid morphology with well-demarcated, broad, eosinophilic or amphophilic cytoplasm, with nuclei with fine chromatin, some of them with presence of eosinophilic nucleolus growing in subcutaneous cellular adipose tissue with images of tumor necrosis and vascular invasion. There are 12 mitoses per 10 high-power fields. The tumor is associated with vascular structures with large areas of blood extravasation and hemosiderophages. The tumor infiltrates the included striated muscle tissue and the surgical resection margin. Transition is identified between well-differentiated low-grade areas (benign glomus tumor) and the predominant high-grade tumor. Immunohistochemical profile was: Actin ML (focal), Actin HHF35, H-caldesmon and Collagen IV (characteristic pericellular and diffuse pattern), p16 (predominantly cytoplasmic) positive; CKAE1-AE3, CK19, Myogenin, Desmin, S100, HMB45, MELAN-A, CD34, CD31, ERG, FACTORVIII, CD138, EMA, CD68, TFE3, D2-40 and HHV8 negative. (A) Hematoxylin-eosin (HE) X 10. Peripheral focus of benign glomus tumor. (B) HE X 10. Peripheral focus of benign glomus (larger diameter). (C) IHC X 10. Patchy positive staining for ML-Actin. (D) HE X 20. Solid tumor growth on a collagenous stroma. The cells are of epithelioid morphology with clear or amphophilic cytoplasm very well demarcated and enlarged oval nuclei with fine chromatin. (**E**) HE X 5. Low magnification, in the periphery of the tumor two images of venous vascular invasion and infiltration of the included adipose and striated muscle tissue are observed. (**F**) HE X 20. Venous vascular tumor invasion.

New biopsy attempts were unsuccessful. Surgical excision showed a tumor affecting the wall musculature and presenting an infiltrative, friable, and vascularized appearance, compatible with a diagnosis of malignant glomus tumor. The patient was referred to our hospital, a national reference center for sarcoma, for evaluation of radical surgery. The pathological review of the surgical specimen confirmed the diagnosis of GT (Figure 2).

Molecular biology analysis with Next-Generation Sequencing (NGS, MiSeq Illumina) revealed the presence of a pathogenic variant in the BRAF gene (NM_004333,4): c.1799T>A, p.Val600Glu, (Coverage 1274x, 20.3% allelic frequency). No other pathogenic genetic alteration was identified.

**Figure 3:**
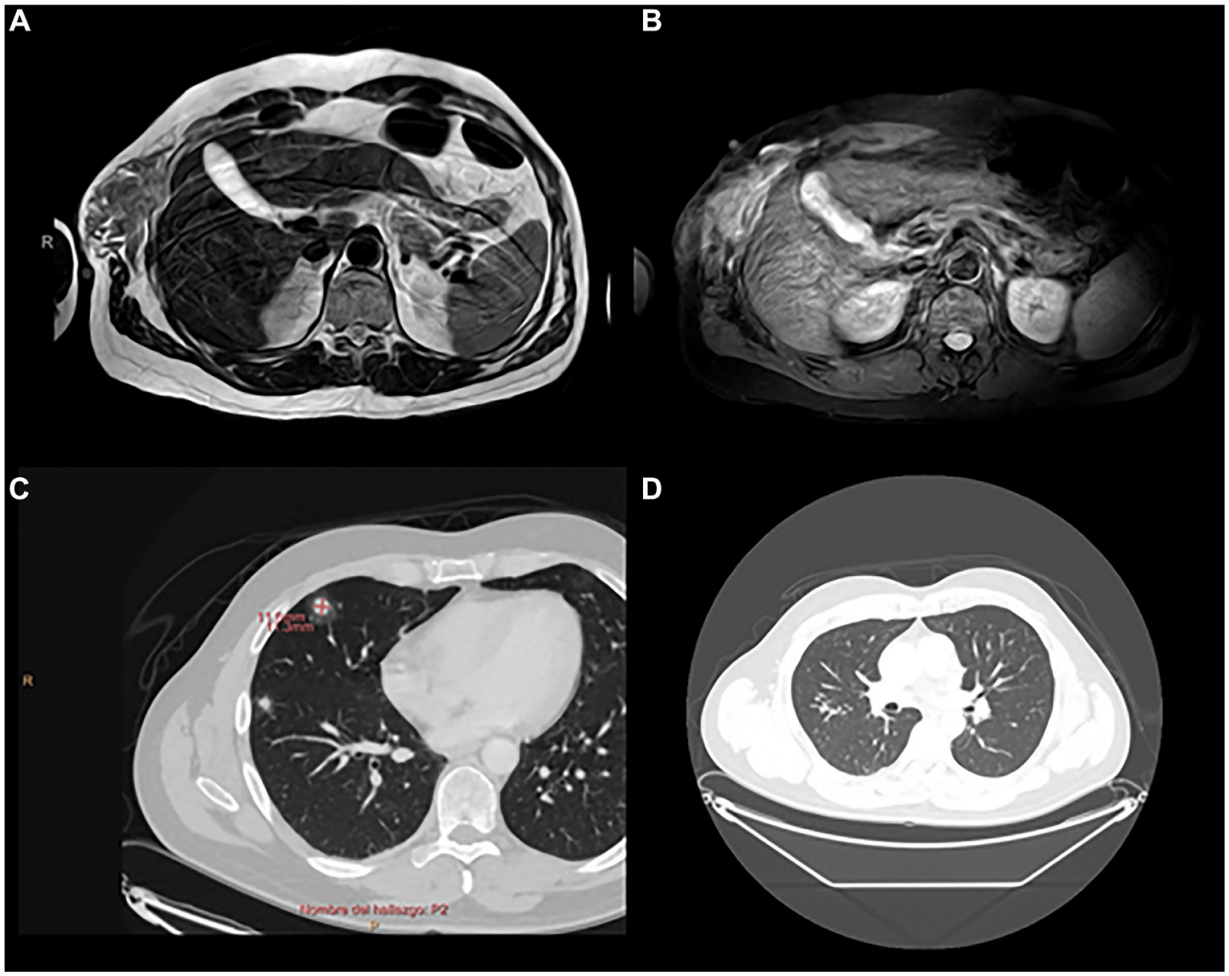
Radiological imaging before treatment. (**A**, **B**) Chest MRI 07/13/2021. Locally aggressive chest wall mass (85 × 50 x 50 mm) with intercostal extension between 7th and 9th arches and bone and muscle invasion. Fat-suppressed MRI sequence shows bone marrow edema in the contiguous 8th costal arch secondary to tumoral invasion. (**C**, **D**) CT scan 08/03/2021. Findings are suggestive of pulmonary metastatic involvement. Pulmonary tumor embolism, *tree-in-bud* pattern associated with a subsegmental artery in the right upper lobe.

Although further surgery to widen surgical margins was recommended, there was rapid tumor growth at the surgical site, with disabling local pain. In addition, the patient presented with marked clinical deterioration consisting of progressive weight loss and profuse sweating. Chest MRI and body CT scan revealed a 6.4 cm tumor mass with local invasion of the 7th and 8th ribs, lung metastases with lung tumor embolisms, liver metastasis, and right iliac bone metastases (Figure 3).

**Figure 4:**
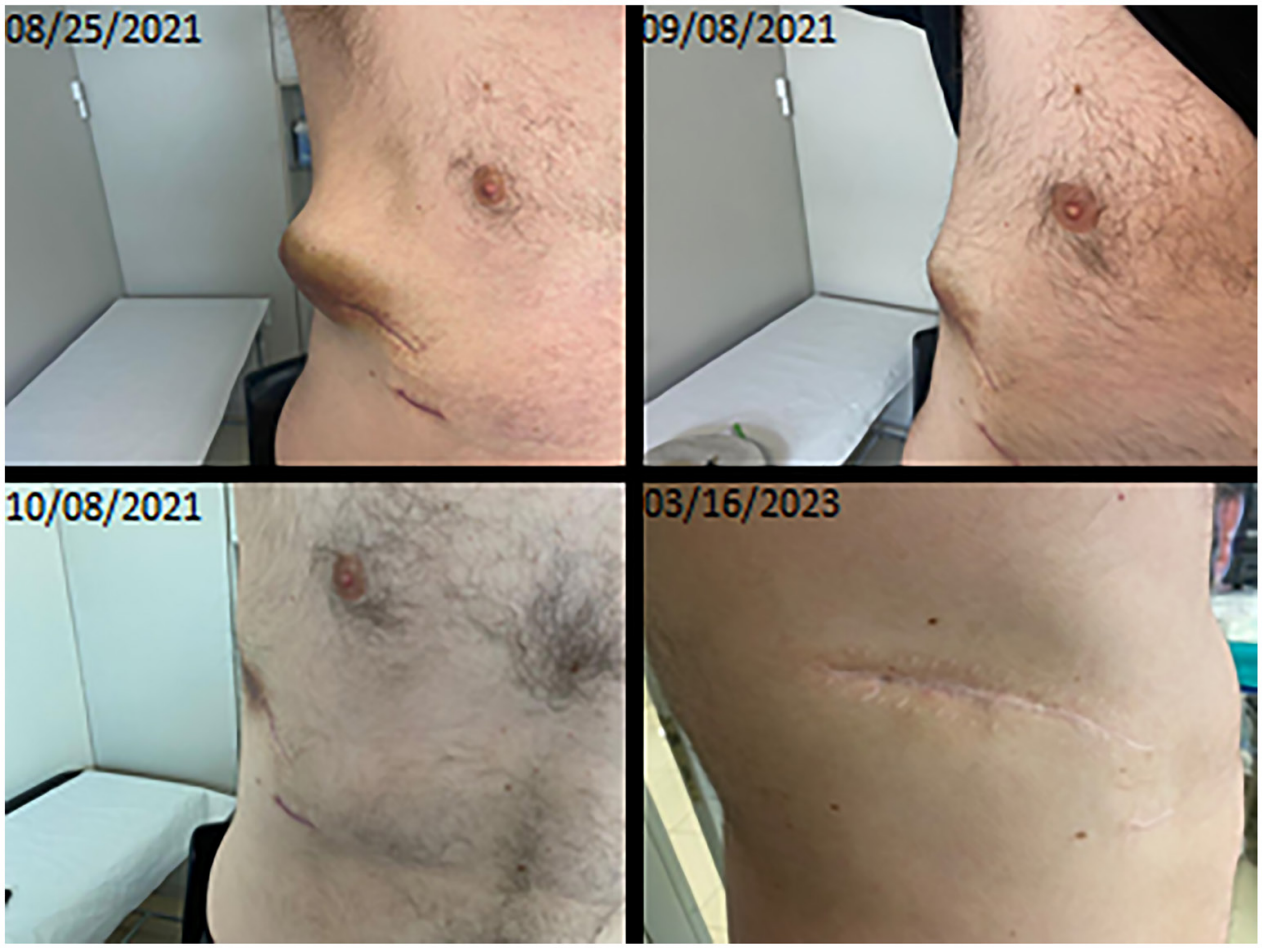
Clinical evolution of the lesion after initiation of targeted treatment.

Given the absence of standard systemic treatment in stage IV malignant glomus tumors, we considered including the patient in a phase I study with a BRAF inhibitor. However, the patient was found ineligible due to a recent onset grade 4 hypertransaminasemia in probable relation to his oncologic disease. Treatment with the BRAF inhibitor encorafenib (450 mg once a day) in combination with the MEK inhibitor binimetinib (45 mg twice daily) was initiated upon compassionate use authorization. The patient experienced rapid clinical improvement together with a dramatic complete response of the right chest soft tissue mass, within 6 weeks of treatment initiation (Figure 4). Liver function (Grade 4 ALT/AST elevation), returned to normal limits after only after two weeks of treatment.

**Figure 5:**
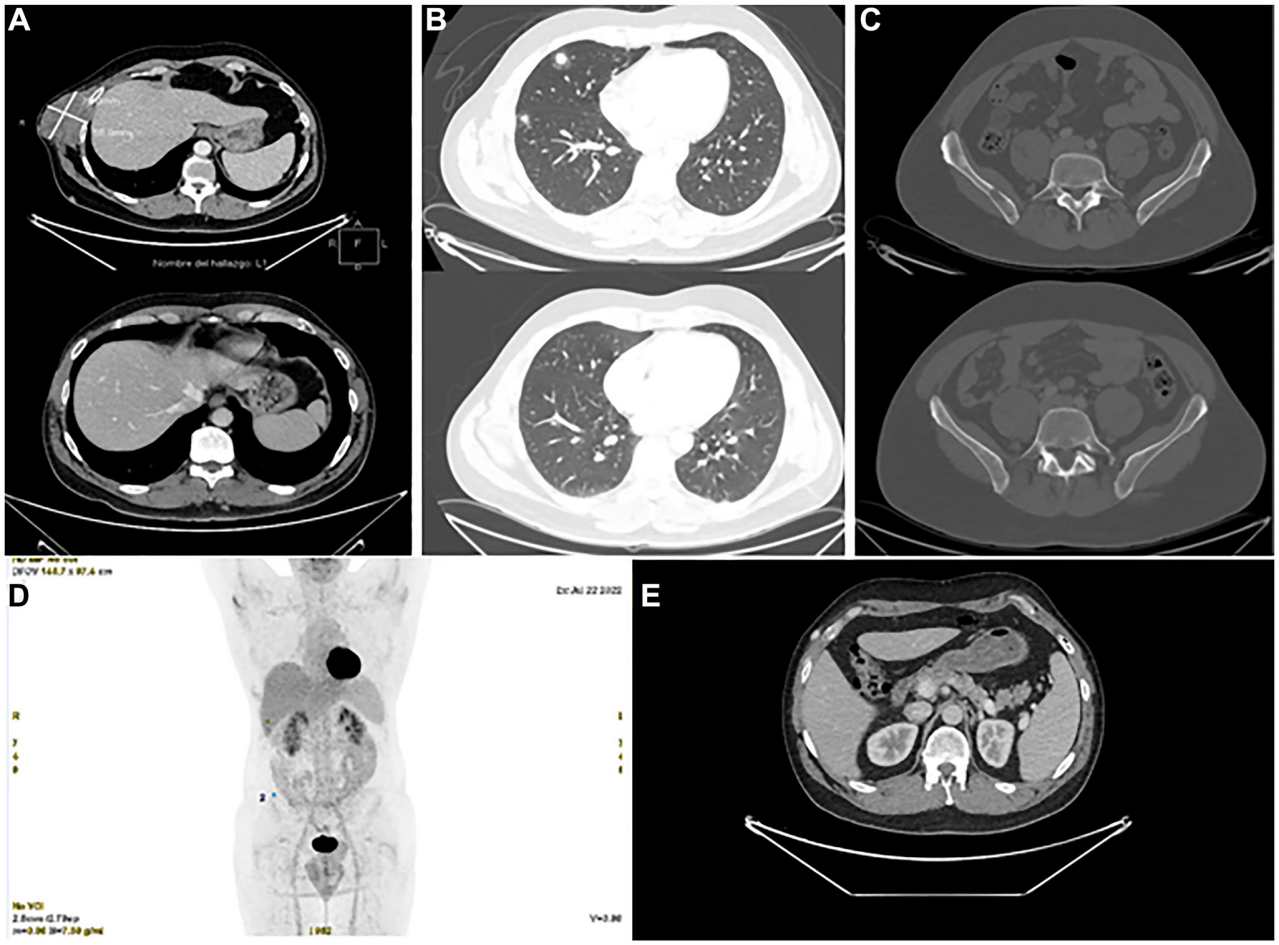
Radiological evolution after targeted therapy. Comparison of CT scans with intravenous contrast, baseline vs. after 6 months of treatment (**A**–**C**) and metabolic complete response on CT-PET (D). (A) Complete radiological response of right costal mass, persistence of benign hypervascular component. (B) Complete radiological response of metastatic pulmonary nodules. (C) Complete radiologic response of right iliac lesion. (**D**) Metabolic complete response in CT–PET 07/20/2022. (**E**) Persistence of benign hypervascular subcutaneous, intermuscular and intramuscular nodules adjacent to the primary tumor in the right lateral abdominal wall.

At the most recent follow-up in August 2024, the patient remains on a complete metabolic and radiologic response for more than two years since encorafenib plus binimetinib was started (Figure 5). The patient has neither presented any treatment-related side effects nor has required dose interruption or modification, and remains completely asymptomatic.

## DISCUSSION

Glomus tumors are perivascular mesenchymal neoplasms that arise from structures called glomus bodies. According to their histological characteristics, they are classified as benign, glomus tumors of uncertain malignant potential (GT-UMP), or malignant [1, 2].

The genetic basis of glomus tumors is yet to be fully elucidated; however, BRAF V600E mutations have been reported in approximately 6–11% of sporadic cases [3, 4]. An exhaustive review of reported GT-UMP and malignant GT harboring BRAF V600E mutation is available in Table 1. Chakrapani et al. [3] also identified three GTs harboring the BRAF V600E mutation in a series of 28 patients with a GT diagnosis. Interestingly, their study did not find an apparent correlation between mutational status and the Ki-67 index, and they reported no differences in histological features between mutated and non-mutated GTs [3]. The largest published series, reported by Karamzadeh et al. [4], included 102 cases of glomus tumors (56% benign, 15% GT-UMP, and 29% malignant). BRAF V600E mutations were detected in six tumors (6%), all of which were classified as malignant glomus tumors or GT-UMP, suggesting that BRAF mutations could be associated with a more aggressive biology. Two of five patients with BRAF V600E-mutated tumors where follow-up information was available had progressive disease: 1 GT-UMP experimenting local recurrence and 1 malignant GT with enlarging residual disease [4]. Chang et al. [6] described the case of a rapidly growing and spontaneously hemorrhagic GT-UMP in the orbit that required urgent orbitotomy and excision of the hemorrhagic tumor. Histopathologic examination of surgical specimen revealed increased mitotic activity, focal spindle morphology, partial necrosis, areas of hypercellularity, and prominent branching capillary-type vessels surrounding the tumor cells. A BRAF V600E mutation was detected by specific immunohistochemistry testing [6]. Dahlin et al. also reported the case of an intraneural GT-UMP involving the median nerve [7], where surgical resection of the median nerve with macroscopically free margins was performed, with no signs of recurrence reported 2 years after surgery. A BRAF V600E mutation was found in an NGS-based analysis of the surgical specimen [7]. Cuviello et al. [5] recently reported a patient with a localized BRAF V600E-mutant malignant GT of the right brachial plexus, which responded positively to BRAF/MEK targeting drugs (dabrafenib 150 mg twice daily plus trametinib 2 mg daily). The patient experienced a decrease in tumor size over the first 9 months and normalization of extremity function. This was the first report of successful BRAF/MEK inhibition treatment for a BRAF V600E-mutant GT, noting that systemic treatment was used to avoid aggressive surgical resection that could have potentially led to significant neurologic deficits [5].

**Table 1:** Review of available case reports of malignant GT or GT-UMP harboring BRAF V600E mutation

Case No.	Reference (author, year)	Classification	Location	Staging	Treatment	Status at time of publication
1.	Chakrapani, 2012 [3]	Unknown	Distal leg	Localized	Surgery	Local recurrence after 3 years
2.		Unknown	Thigh	Localized	Surgery	Alive, no evidence of recurrence
3.		Unknown	Upper arm	Localized	Surgery	Alive, no evidence of recurrence
4.	Dahlin, 2016 [7]	GT-UMP	Median nerve	Localized	Surgery	Alive, no evidence of recurrence
5.	Karamzadeh, 2017 [4]	GT-UMP	Parapharyngeal	Localized	Surgery	Alive, no evidence of recurrence
6.		GT-UMP	Ankle	Localized	Surgery	Alive, no evidence of recurrence
7.		GT-UMP	Thigh	Localized	Surgery	Local recurrence at 1, 3 and 10 years
8.		Malignant GT	Foot	Localized	Surgery	Alive, no evidence of recurrence
9.		Malignant GT	Brachial plexus	Localized	Surgery	Alive, residual slowly enlarging tumor
10.		Malignant GT	Hand	Localized	Surgery	Unknown
11.	Cuviello, 2018 [5]	Malignant GT	Brachial plexus	Localized	Systemic targeted therapy	Alive, maintained response
12.	Chang, 2023 [6]	GT-UMP	Orbit	Localized	Surgery	Alive, no evidence of recurrence

Our report underscores the importance of comprehensive tumor genotyping to identify potentially actionable targets in rare, orphan tumors such as soft tissue sarcomas (STS), a heterogeneous group of tumors for which therapeutic options are often limited. Gounder et al. [9] described that the systematic implementation of NGS in STS reported clinical benefits, with approximately 5% of patients experiencing a change in their final diagnosis or treatment [8]. In this context, the detection of the BRAF V600E mutation opens the door to an agnostic approach based on targeted therapies, such as BRAF inhibitors, which are effective in other malignancies like melanoma, colorectal cancer, and certain thyroid tumors [9].

RAF kinase inhibitors used to reduce MAP kinase signaling pathway activation, were first studied in clinical trials involving patients with melanoma. Encorafenib, a BRAF inhibitor, and binimetinib, a MEK inhibitor, are two orally bioavailable drugs. In June 2018, they both received their first approval in the United States for their combined use in patients with unresectable or metastatic melanoma with a *BRAF V600E* or -*V600K* mutation [10]. Combination therapy of encorafenib and binimetinib plus cetuximab has also recently been tested for the treatment of metastatic colorectal cancer with positive results [11].

Class I BRAF mutations represent a recognized agnostic treatment opportunity. The combination of a BRAF V600E inhibitor plus a downstream MEK inhibitor avoids paradoxical activation of the MAPK pathway in normal tissues, reducing the risk of skin tumors while enhancing pathway inhibition within *BRAF* mutant tumors [12, 13]. This achieves better clinical responses, as confirmed in melanoma and NSCLC studies [13, 14]. A similar approach can be recommended for extremely rare conditions such as malignant GT. Food and Drugs Administration (FDA) agnostic approval of dabrafenib and trametinib for BRAF V600E mutant solid tumors on 22nd June 2022 opens the door to targeted therapy in this subset of patients [15], including rare cancers as malignant GT, where other therapeutic options could not be effective or available.

## CONCLUSIONS

This case report highlights the importance of full tumor genotyping to identify potentially actionable targets in rare tumors such as malignant glomus tumors.

BRAF V600E mutations represent a recognized agnostic treatment opportunity, and the combination of a BRAF inhibitor and a downstream MEK inhibitor can be an effective treatment option in such cases.

To our knowledge, our patient represents the first reported case of a metastatic malignant GT successfully treated with BRAF and MEK inhibitors, achieving a long-lasting complete morpho-metabolic response.

## References

[1] WHO Classification of Tumours Editorial Board. Soft tissue and bone tumours. Lyon (France): International Agency for Research on Cancer; 2020. (WHO classification of tumours series, 5th ed. Vol. 3). https://publications.iarc.fr/588.

[2] Folpe AL , Fanburg-Smith JC , Miettinen M , Weiss SW . Atypical and malignant glomus tumors: analysis of 52 cases, with a proposal for the reclassification of glomus tumors. Am J Surg Pathol. 2001; 25:1–12. 10.1097/00000478-200101000-00001. 11145243

[3] Chakrapani A , Warrick A , Nelson D , Beadling C , Corless CL . BRAF and KRAS mutations in sporadic glomus tumors. Am J Dermatopathol. 2012; 34:533–35. 10.1097/DAD.0b013e31823931b4. 22317887

[4] Karamzadeh Dashti N , Bahrami A , Lee SJ , Jenkins SM , Rodriguez FJ , Folpe AL , Boland JM . BRAF V600E Mutations Occur in a Subset of Glomus Tumors, and Are Associated With Malignant Histologic Characteristics. Am J Surg Pathol. 2017; 41:1532–41. 10.1097/PAS.0000000000000913. 28834810

[5] Cuviello A , Goyal A , Zick A , Ahlawat S , Rodriguez FJ , Belzberg AJ , Pratilas CA . Sporadic Malignant Glomus Tumor of the Brachial Plexus With Response to Targeted Therapy Directed Against Oncogenic *BRAF* . JCO Precis Oncol. 2018; 2018:PO.17.00261. 10.1200/PO.17.00261. 30556047 PMC6292440

[6] Chang EK , Chiou CA , Lefebvre DR , Stagner AM . A Rapidly Expanding Hemorrhagic BRAF-Mutant Orbital Atypical Glomus Tumor. Ophthalmic Plast Reconstr Surg. 2024; 40:e11–14. 10.1097/IOP.0000000000002513. 37695168

[7] Dahlin LB , Scherman P , Besjakov J , Lindberg E , Solomon DA , Horvai AE , Perry A . Intraneural glomus tumor of “uncertain malignant potential” and with BRAF mutation in the median nerve - an unusual case. Clin Neuropathol. 2017; 36:164–70. 10.5414/NP300989. 28438258

[8] Lin J , Luo J , Chen H , Li Z , Shi Y , Sun H , Zhang Z , Zhou G , Zhong D . Clinicopathologic features and BRAF mutation status of tracheal glomus tumors - Characterization of 4 cases and the distinction from low-grade neuroendocrine tumors. Ann Diagn Pathol. 2021; 55:151797. 10.1016/j.anndiagpath.2021.151797. 34509154

[9] Gounder MM , Ali SM , Robinson V , Bailey M , Ferraro R , Patel NM , Krishnan A , Millis SZ , Dickson MA , D’Angelo SP , Keohan ML , Miller VA , Schwartz GK , et al. Impact of next-generation sequencing (NGS) on diagnostic and therapeutic options in soft-tissue and bone sarcoma. J Clin Oncol. 2017; 35:11001. 10.1200/JCO.2017.35.15_suppl.11001.

[10] Shirley M . Encorafenib and Binimetinib: First Global Approvals. Drugs. 2018; 78:1277–84. 10.1007/s40265-018-0963-x. 30117021

[11] Tabernero J , Grothey A , Van Cutsem E , Yaeger R , Wasan H , Yoshino T , Desai J , Ciardiello F , Loupakis F , Hong YS , Steeghs N , Guren TK , Arkenau HT , et al. Encorafenib Plus Cetuximab as a New Standard of Care for Previously Treated *BRAF* V600E-Mutant Metastatic Colorectal Cancer: Updated Survival Results and Subgroup Analyses from the BEACON Study. J Clin Oncol. 2021; 39:273–84. 10.1200/JCO.20.02088. 33503393 PMC8078423

[12] Lito P , Pratilas CA , Joseph EW , Tadi M , Halilovic E , Zubrowski M , Huang A , Wong WL , Callahan MK , Merghoub T , Wolchok JD , de Stanchina E , Chandarlapaty S , et al. Relief of profound feedback inhibition of mitogenic signaling by RAF inhibitors attenuates their activity in BRAFV600E melanomas. Cancer Cell. 2012; 22:668–82. 10.1016/j.ccr.2012.10.009. 23153539 PMC3713778

[13] Flaherty KT , Infante JR , Daud A , Gonzalez R , Kefford RF , Sosman J , Hamid O , Schuchter L , Cebon J , Ibrahim N , Kudchadkar R , Burris HA 3rd , Falchook G , et al. Combined BRAF and MEK inhibition in melanoma with BRAF V600 mutations. N Engl J Med. 2012; 367:1694–703. 10.1056/NEJMoa1210093. 23020132 PMC3549295

[14] Planchard D , Smit EF , Groen HJM , Mazieres J , Besse B , Helland Å , Giannone V , D’Amelio AM Jr , Zhang P , Mookerjee B , Johnson BE . Dabrafenib plus trametinib in patients with previously untreated BRAF^V600E^-mutant metastatic non-small-cell lung cancer: an open-label, phase 2 trial. Lancet Oncol. 2017; 18:1307–16. 10.1016/s1470-2045(17)30679-4. 28919011

[15] U. S. Food and Drug Administration. FDA grants accelerated approval to dabrafenib in combination with trametinib for unresectable or metastatic solid tumors. Silver Spring (MD): FDA; 2022. https://www.fda.gov/drugs/resources-information-approved-drugs/fda-grants-accelerated-approval-dabrafenib-combination-trametinib-unresectable-or-metastatic-solid#:~:text=On%20June%2022%2C%202022%2C%20the,BRAF%20V600E%20mutation%20who%20have

